# Supplementary figures and images for: Microbial signature profiles of Penaeus vannamei larvae in low-survival hatchery tanks affected by vibriosis
Source: PeerJ. 2023 Sep 1;11:e15795. doi: 10.7717/peerj.15795 (PMC10476614; doi:10.7717/peerj.15795)

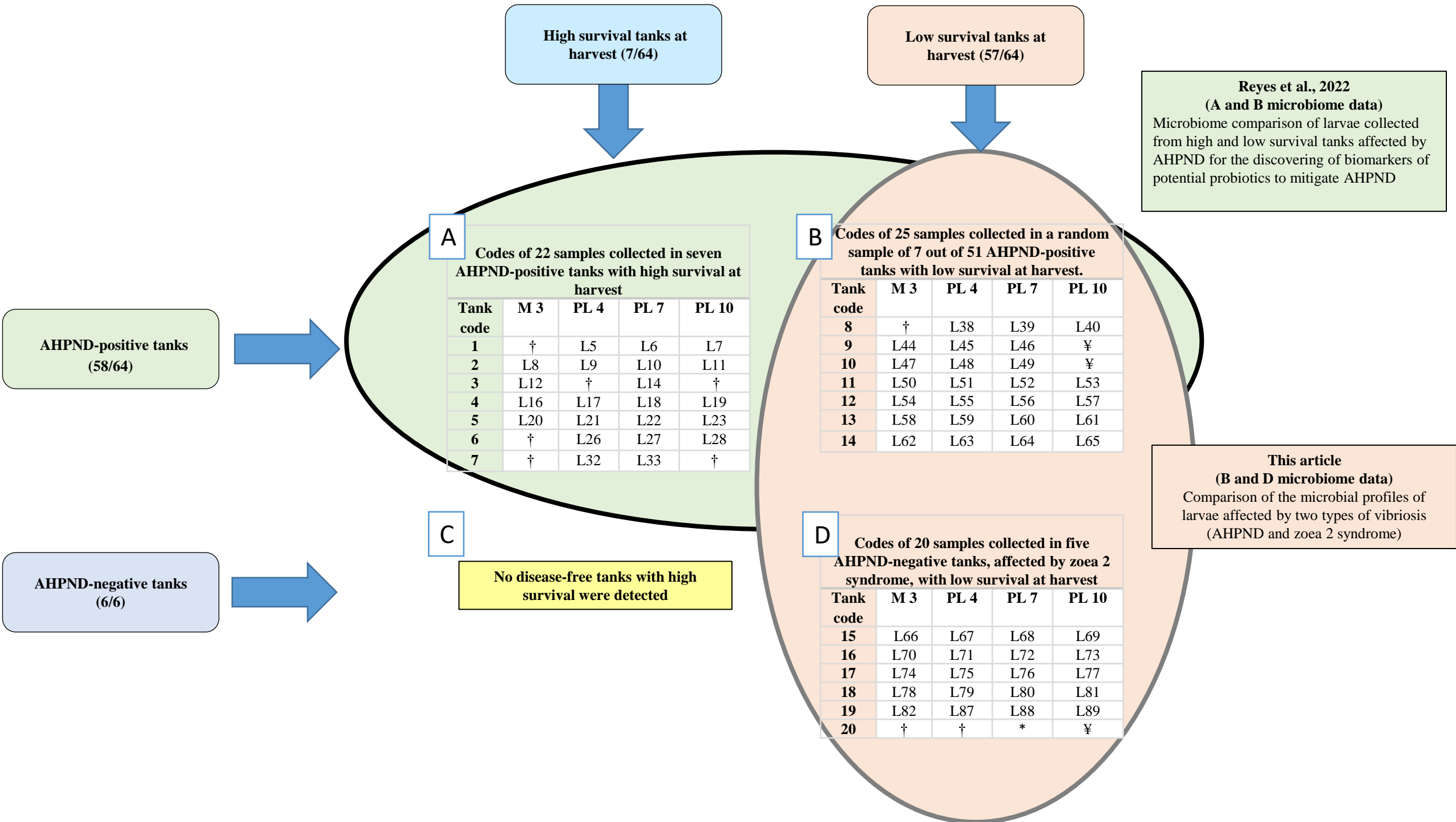

Supplement: Supplemental Information 1 — An observational study was conducted in commercial hatchery tanks (64 tanks, 35 tons, South America) to investigate the microbiome of P. vannamei larvae naturally affected by vibriosis. A total of 58 tanks were AHPND-positive and six were AHPND-negative. A previous study compared the microbiome of larvae collected from (A) high (22 samples from 7 tanks) and (B) low (25 samples from 7 tanks randomly selected from 51 tanks) survival tanks affected by AHPND, for future development of probiotics to mitigate the disease, A and B (Reyes et al., 2022). (C) No disease-free tanks with high survival were detected. In the present study, we analyzed the microbiome of larvae affected by zoea 2 syndrome (D) (20 samples from five tanks that were AHPND negative) and reused the microbiome data of the 25 samples of AHPND-affected larvae from low-survival tanks (B) with the new goal of comparing the microbial profiles of larvae affected by two types of vibriosis (AHPND and zoea 2 syndrome), B and D. One sample of each of the following stages was collected from each one of the 12 tanks: Mysis 3 (M3), Postlarvae 4 (PL4), Postlarvae 7 (PL7), and Postlarvae 10 (PL10), except in two of the seven tanks affected by AHPND, where only samples for M3, PL4, and PL7 were collected in each of the two tanks because the populations died at the PL9 stage (¥). One sample from a third tank affected by AHPND did not pass the DNA quality control ( † ). Sample not used for the analysis (*). [file peerj-11-15795-s001.pdf]

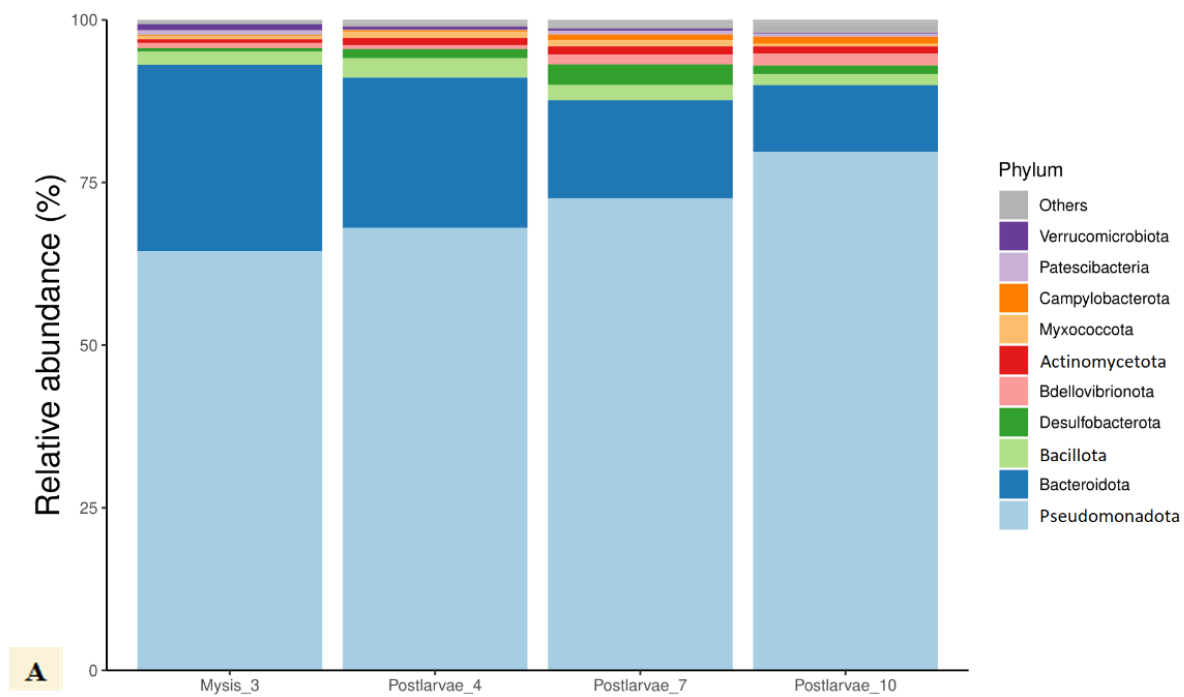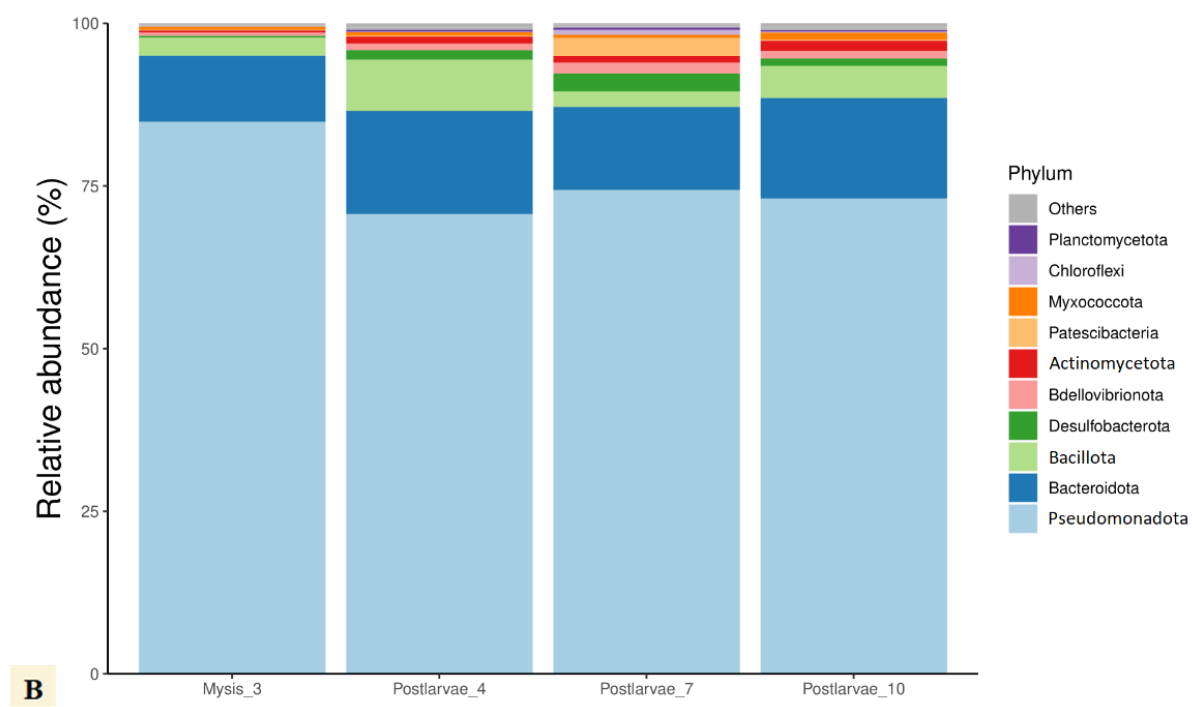

Supplement: Supplemental Information 2 — A) Shannon index for AHPND-affected and zoea 2 syndrome-affected tanks. (B) ACE index for AHPND-affected and zoea 2 syndrome-affected tanks. For each survival condition, ns indicates the alpha diversity index is not significantly different at P < 0.05. [file peerj-11-15795-s002.pdf]

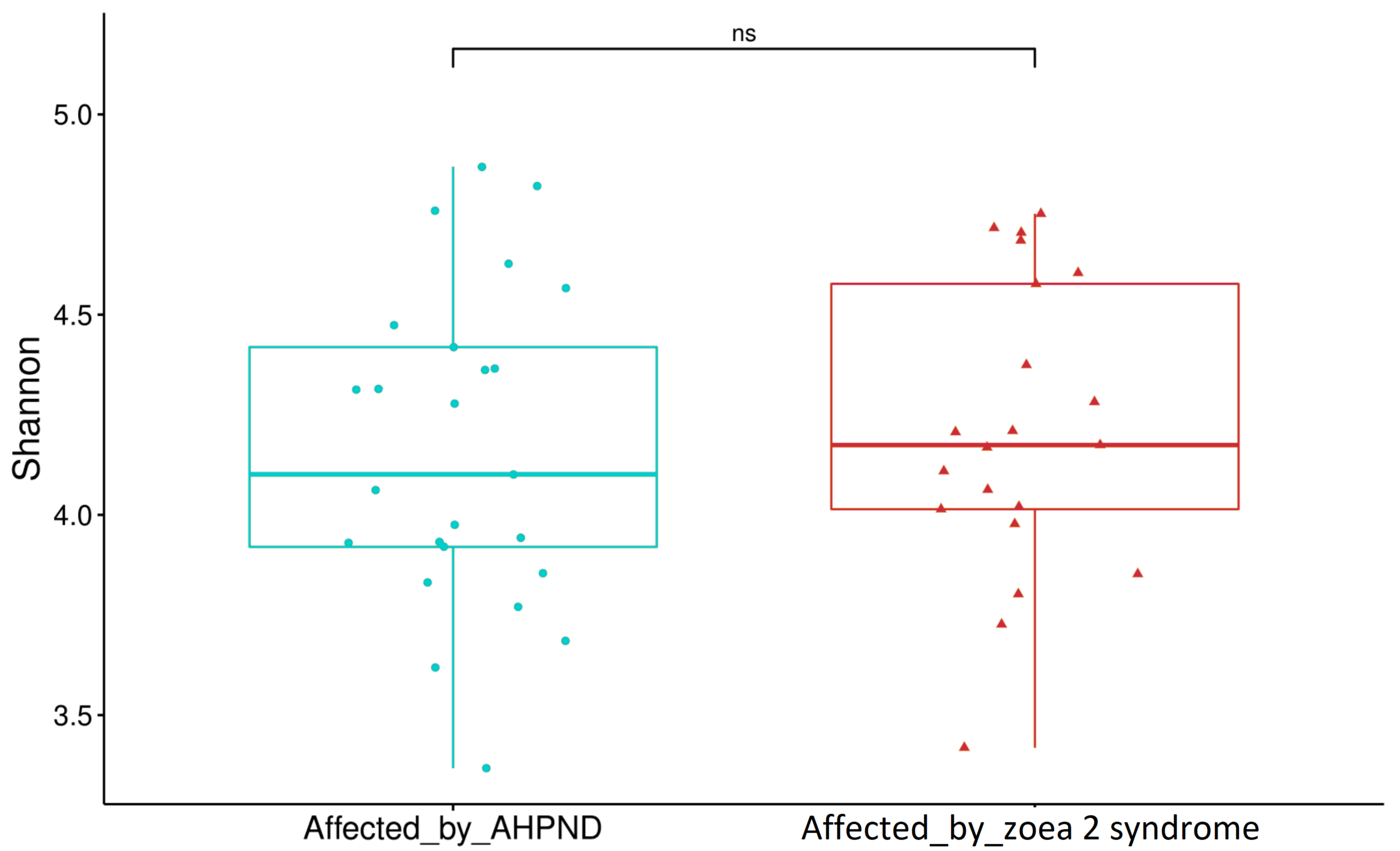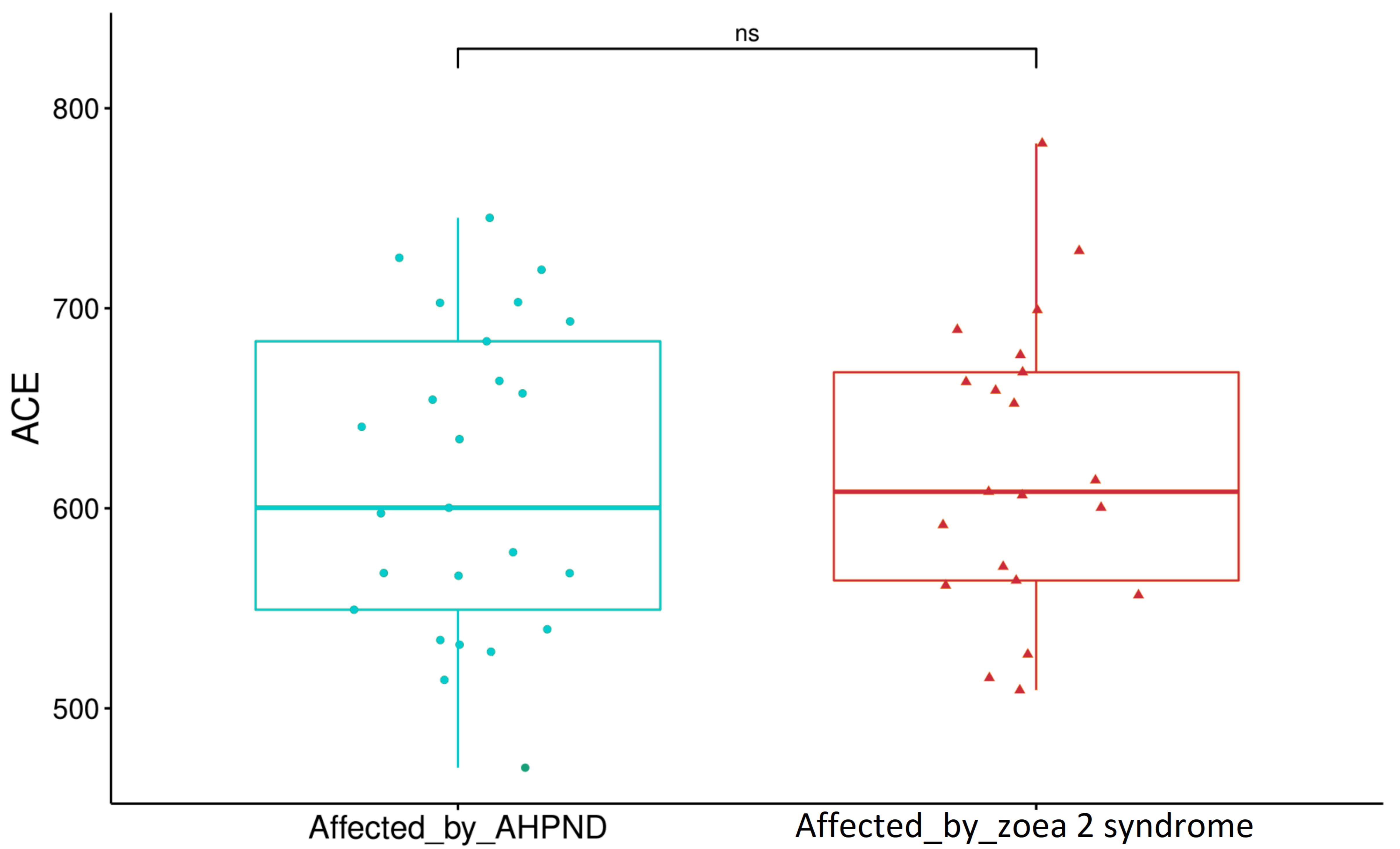

Supplement: Supplemental Information 3 — (A) tanks affected by AHPND and (B) tanks affected by zoea 2 syndrome. [file peerj-11-15795-s003.pdf]
