# Supplementary material for: Microbial signature profiles of Penaeus vannamei larvae in low-survival hatchery tanks affected by vibriosis
Source: PeerJ. 2023 Sep 1;11:e15795. doi: 10.7717/peerj.15795 (PMC10476614; doi:10.7717/peerj.15795)
Supplement: Supplemental Information 6 — * Microbiome data from the low-survival AHPND-affected tanks published by Reyes et al., 2022. [file peerj-11-15795-s006.docx]

| **Disease**  **condition** | **Tank code** | **Survival at harvest (%)** | **Sample code** | **Larval stage** | **Number of ASVs** | **Number of clean reads** | **Good’s coverage (%)** | **Error rate (%)** | **GC content (%)** | **Phred Q30 (%)** |
| --- | --- | --- | --- | --- | --- | --- | --- | --- | --- | --- |
| Affected by AHPND | 8* | 25.0 | L38 | Postlarvae 4 | 349 | 145,592 | 99.999 | 0.03 | 53.83 | 88.15 |
|  |  |  | L39 | Postlarvae 7 | 358 | 160,587 | 99.999 | 0.03 | 52.39 | 88.48 |
|  |  |  | L40 | Postlarvae 10 | 360 | 146,752 | 99.995 | 0.04 | 54.06 | 85.25 |
|  | 9* | 0.0 | L44 | Mysis 3 | 309 | 130,041 | 99.996 | 0.03 | 53.87 | 88.31 |
|  |  |  | L45 | Postlarvae 4 | 399 | 136,451 | 99.998 | 0.03 | 53.10 | 88.94 |
|  |  |  | L46 | Postlarvae 7 | 397 | 147,445 | 99.996 | 0.03 | 54.41 | 88.79 |
|  | 10* | 0.0 | L47 | Mysis 3 | 331 | 147,831 | 99.999 | 0.03 | 52.26 | 88.28 |
|  |  |  | L48 | Postlarvae 4 | 445 | 151,411 | 99.998 | 0.03 | 54.04 | 89.05 |
|  |  |  | L49 | Postlarvae 7 | 482 | 160,061 | 99.997 | 0.04 | 52.84 | 84.44 |
|  | 11* | 25.0 | L50 | Mysis 3 | 347 | 141,911 | 99.999 | 0.03 | 53.27 | 88.46 |
|  |  |  | L51 | Postlarvae 4 | 431 | 143,408 | 100.000 | 0.03 | 54.59 | 88.58 |
|  |  |  | L52 | Postlarvae 7 | 435 | 153,395 | 99.998 | 0.04 | 52.90 | 84.50 |
|  |  |  | L53 | Postlarvae 10 | 466 | 167,189 | 99.994 | 0.03 | 53.29 | 88.39 |
|  | 12* | 25.0 | L54 | Mysis 3 | 370 | 134,186 | 99.999 | 0.03 | 53.72 | 88.46 |
|  |  |  | L55 | Postlarvae 4 | 437 | 157,179 | 99.997 | 0.03 | 54.76 | 88.99 |
|  |  |  | L56 | Postlarvae 7 | 452 | 139,258 | 99.996 | 0.03 | 53.79 | 88.71 |
|  |  |  | L57 | Postlarvae 10 | 375 | 133,245 | 99.998 | 0.03 | 53.69 | 88.99 |
|  | 13* | 25.0 | L58 | Mysis 3 | 376 | 140,622 | 99.999 | 0.03 | 54.14 | 88.83 |
|  |  |  | L59 | Postlarvae 4 | 447 | 146,989 | 100.000 | 0.04 | 52.21 | 85.16 |
|  |  |  | L60 | Postlarvae 7 | 381 | 130,641 | 99.998 | 0.04 | 52.03 | 85.08 |
|  |  |  | L61 | Postlarvae 10 | 443 | 139,971 | 99.999 | 0.04 | 52.27 | 85.10 |
|  | 14* | 25.0 | L62 | Mysis 3 | 342 | 138,007 | 99.995 | 0.03 | 53.57 | 88.94 |
|  |  |  | L63 | Postlarvae 4 | 412 | 136,721 | 99.998 | 0.03 | 54.07 | 89.00 |
|  |  |  | L64 | Postlarvae 7 | 407 | 134,369 | 99.999 | 0.03 | 53.88 | 88.83 |
|  |  |  | L65 | Postlarvae 10 | 436 | 139,961 | 99.996 | 0.03 | 54.41 | 89.17 |
| Affected by zoea 2 syndrome | 15 | 56.7 | L66 | Mysis 3 | 369 | 140,344 | 99.992 | 0.03 | 53.61 | 88.46 |
|  |  |  | L67 | Postlarvae 4 | 462 | 135,116 | 99.998 | 0.03 | 54.19 | 88.36 |
|  |  |  | L68 | Postlarvae 7 | 427 | 138,704 | 99.995 | 0.03 | 53.37 | 88.29 |
|  |  |  | L69 | Postlarvae 10 | 194 | 141,895 | 99.991 | 0.03 | 53.65 | 88.85 |
|  | 16 | 47.5 | L70 | Mysis 3 | 385 | 168,831 | 99.997 | 0.03 | 53.47 | 88.83 |
|  |  |  | L71 | Postlarvae 4 | 462 | 164,378 | 99.998 | 0.03 | 53.25 | 88.71 |
|  |  |  | L72 | Postlarvae 7 | 387 | 142,897 | 99.992 | 0.03 | 53.81 | 88.96 |
|  |  |  | L73 | Postlarvae 10 | 429 | 158,249 | 99.995 | 0.03 | 54.49 | 88.74 |
|  | 17 | 25.0 | L74 | Mysis 3 | 182 | 159,871 | 99.988 | 0.03 | 54.78 | 88.11 |
|  |  |  | L75 | Postlarvae 4 | 397 | 132,629 | 99.99 | 0.03 | 54.47 | 87.52 |
|  |  |  | L76 | Postlarvae 7 | 372 | 161,793 | 99.995 | 0.03 | 54.28 | 89.21 |
|  |  |  | L77 | Postlarvae 10 | 419 | 158,028 | 99.993 | 0.03 | 53.23 | 88.16 |
|  | 18 | 39.2 | L78 | Mysis 3 | 368 | 157,671 | 99.995 | 0.03 | 53.65 | 88.21 |
|  |  |  | L79 | Postlarvae 4 | 448 | 135,876 | 99.991 | 0.03 | 53.88 | 88.14 |
|  |  |  | L80 | Postlarvae 7 | 402 | 158,211 | 99.993 | 0.03 | 53.74 | 88.17 |
|  |  |  | L81 | Postlarvae 10 | 280 | 138,367 | 99.997 | 0.03 | 54.91 | 87.24 |
|  | 19 | 0.0 | L82 | Mysis 3 | 351 | 140,841 | 99.994 | 0.03 | 54.32 | 88.37 |
|  |  |  | L87 | Postlarvae 4 | 443 | 139,252 | 99.997 | 0.03 | 54.27 | 88.56 |
|  |  |  | L88 | Postlarvae 7 | 416 | 150,863 | 99.998 | 0.03 | 54.63 | 88.63 |
|  |  |  | L89 | Postlarvae 10 | 453 | 157,926 | 99.996 | 0.03 | 54.16 | 88.38 |
