# Supplementary material for: Microbial signature profiles of Penaeus vannamei larvae in low-survival hatchery tanks affected by vibriosis
Source: PeerJ. 2023 Sep 1;11:e15795. doi: 10.7717/peerj.15795 (PMC10476614; doi:10.7717/peerj.15795)
Supplement: Supplemental Information 7 [file peerj-11-15795-s007.docx]

| **Disease condition** | **Stage** | **Phylum** | | |  | **Family** | | | |  | **Genera** | | | | |  |
| --- | --- | --- | --- | --- | --- | --- | --- | --- | --- | --- | --- | --- | --- | --- | --- | --- |
|  |  | Pseudomonadota | Bacteroidota | Bacillota | **Percentage (%)** | Rhodobacteraceae | Vibrionaceae | Flavobacteriaceae | Bacillaceae | **Percentage (%)** | *Marinibacterium* | *Catenococcus* | *Vibrio* | *Gilvibacter* | *Bacillus* | **Percentage (%)** |
| Affected by AHPND | M3 | 66 | 29 | 2 | 97 | 41 | 11 | 24 | 2 | 78 | 11 | 7 | 4 | 18 | 2 | 42 |
|  | PL4 | 68 | 23 | 3 | 94 | 40 | 8 | 14 | 3 | 65 | 18 | 5 | 3 | 8 | 3 | 37 |
|  | PL7 | 73 | 15 | 2 | 90 | 36 | 12 | 7 | 1 | 56 | 13 | 8 | 3 | 2 | 1 | 27 |
|  | PL10 | 80 | 10 | 2 | 92 | 38 | 23 | 4 | 1 | 66 | 7 | 11 | 11 | 1 | 1 | 31 |
| Affected by zoea 2 syndrome | M3 | 86 | 10 | 2 | 98 | 28 | 49 | 8 | 2 | 87 | 3 | 27 | 21 | 3 | 2 | 56 |
|  | PL4 | 72 | 16 | 8 | 96 | 44 | 4 | 10 | 7 | 65 | 22 | 2 | 2 | 1 | 7 | 34 |
|  | PL7 | 75 | 13 | 2 | 90 | 35 | 22 | 8 | 1 | 66 | 22 | 15 | 6 | 0 | 1 | 44 |
|  | PL10 | 75 | 16 | 3 | 94 | 35 | 17 | 12 | 3 | 67 | 15 | 6 | 11 | 0 | 3 | 35 |
