# Supplementary material for: Microbial signature profiles of Penaeus vannamei larvae in low-survival hatchery tanks affected by vibriosis
Source: PeerJ. 2023 Sep 1;11:e15795. doi: 10.7717/peerj.15795 (PMC10476614; doi:10.7717/peerj.15795)
Supplement: Supplemental Information 8 [file peerj-11-15795-s008.docx]

| **Parameters** | **Larvae affected by AHPND** | **Larvae affected by zoea 2 syndrome** |
| --- | --- | --- |
| Number of nodes | 117 | 120 |
| Number of edges | 557 | 310 |
| Average_degree | 9.5 | 5.16 |
| Average_path_length | 2.3 | 3.18 |
| Network_diameter | 8 | 8 |
| Clustering_coefficient | 0.54 | 0.45 |
| Density | 0.08 | 0.04 |
| Heterogeneity | 0.90 | 0.71 |
| Network centralization | 0.20 | 0.09 |
