# Supplementary material for: Microbial signature profiles of Penaeus vannamei larvae in low-survival hatchery tanks affected by vibriosis
Source: PeerJ. 2023 Sep 1;11:e15795. doi: 10.7717/peerj.15795 (PMC10476614; doi:10.7717/peerj.15795)
Supplement: Supplemental Information 9 [file peerj-11-15795-s009.docx]

| **Disease condition** | **ASV** | **Phylum** | **Class** | **Order** | **Family** | **Genus** | **Species** |
| --- | --- | --- | --- | --- | --- | --- | --- |
| Affected by AHPND | 17 | *Pseudomonadota* | *Alphaproteobacteria* | *Rhodobacterales* | *Rhodobacteraceae* | - | - |
|  | 22 | *Pseudomonadota* | *Alphaproteobacteria* | *Sneathiellales* | *Sneathiellaceae* | *Sneathiella* | - |
|  | 24 | *Bacteroidota* | *Bacteroidia* | *Cytophagales* | *Cyclobacteriaceae* | *Cyclobacterium* | *marinum* |
|  | 26 | *Bacteroidota* | *Bacteroidia* | *Chitinophagales* | *Saprospiraceae* | - | - |
|  | 47 | *Pseudomonadota* | *Gammaproteobacteria* | - | - | - | - |
|  | 51 | *Pseudomonadota* | *Alphaproteobacteria* | *Rhodobacterales* | *Rhodobacteraceae* | - | - |
|  | 62 | *Pseudomonadota* | *Gammaproteobacteria* | *Pseudomonadales* | *Halieaceae* | *Haliea* | - |
|  | 65 | *Pseudomonadota* | *Alphaproteobacteria* | *Rhizobiales* | *Methyloligellaceae* | - | - |
|  | 157 | *Bacteroidota* | *Bacteroidia* | *Chitinophagales* | *Saprospiraceae* | *Lewinella* | - |
|  | 207 | *Pseudomonadota* | *Gammaproteobacteria* | *Enterobacterales* | *Vibrionaceae* | *Catenococcus* | - |
| Affected by zoea 2 syndrome | 12 | *Pseudomonadota* | *Gammaproteobacteria* | *Enterobacterales* | *Vibrionaceae* | *Catenococcus* | - |
|  | 19 | *Bacteroidota* | *Bacteroidia* | *Flavobacteriales* | *Flavobacteriaceae* | *Spongiimonas* | - |
|  | 39 | *Bacteroidota* | *Bacteroidia* | *Flavobacteriales* | *Flavobacteriaceae* | *Meridianimaribacter* | *flavus* |
|  | 50 | *Pseudomonadota* | *Gammaproteobacteria* | *Enterobacterales* | *Vibrionaceae* | *Vibrio* | - |
|  | 56 | *Pseudomonadota* | *Gammaproteobacteria* | *Enterobacterales* | *Colwelliaceae* | *Thalassotalea* | - |
|  | 64 | *Pseudomonadota* | *Gammaproteobacteria* | *Enterobacterales* | *Vibrionaceae* | *Vibrio* | - |
|  | 75 | *Pseudomonadota* | *Gammaproteobacteria* | *Enterobacterales* | *Vibrionaceae* | *Vibrio* | - |
|  | 87 | *Pseudomonadota* | *Gammaproteobacteria* | *Enterobacterales* | *Vibrionaceae* | *Catenococcus* | - |
|  | 90 | *Bacteroidota* | *Bacteroidia* | *Flavobacteriales* | *Flavobacteriaceae* | *Tenacibaculum* | - |
|  | 106 | *Pseudomonadota* | *Gammaproteobacteria* | *Enterobacterales* | *Vibrionaceae* | *Vibrio* | - |
|  | 114 | *Pseudomonadota* | *Gammaproteobacteria* | *Enterobacterales* | *Vibrionaceae* | *Vibrio* | - |
|  | 166 | *Pseudomonadota* | *Gammaproteobacteria* | *Enterobacterales* | *Vibrionaceae* | *Vibrio* | - |
