# Supplementary material for: Microbial signature profiles of Penaeus vannamei larvae in low-survival hatchery tanks affected by vibriosis
Source: PeerJ. 2023 Sep 1;11:e15795. doi: 10.7717/peerj.15795 (PMC10476614; doi:10.7717/peerj.15795)
Supplement: Supplemental Information 10 [file peerj-11-15795-s010.docx]

| **Disease condition** | **ASV** | **Bayes Factor BF_10_** | **Error** | **Evidence in favor of the H_1_ alternative hypothesis (different ASV abundance between tanks affected by AHPND and zoea 2 syndrome)** |
| --- | --- | --- | --- | --- |
| Affected by AHPND | 17 | 8,3 | 8,56E-09 | Strong |
|  | 22 | 3,9 | 1,21E-08 | Moderate |
|  | 24 | 29,7 | 5,09E-09 | Strong |
|  | 26 | 14,3 | 9,35E-09 | Strong |
|  | 47 | 25,4 | 6,17E-09 | Strong |
|  | 51 | 308,5 | 9,27E-10 | Strong |
|  | 62 | 15,1 | 9,16E-09 | Strong |
|  | 65 | 4,3 | 8,60E-09 | Moderate |
|  | 157 | 3,3 | 8,95E-05 | Moderate |
|  | 207 | 11,3 | 9,73E-09 | Strong |
| Affected by zoea 2 syndrome | 12 | 44,5 | 2,47E-09 | Strong |
|  | 19 | 237 | 1,05E-09 | Strong |
|  | 39 | 11,3 | 9,73E-09 | Strong |
|  | 50 | 4,4 | 7,06E-09 | Moderate |
|  | 56 | 2,7 | 8,64E-05 | Weak |
|  | 64 | 40,8 | 2,98E-09 | Strong |
|  | 75 | 5,6 | 4,30E-09 | Moderate |
|  | 87 | 14,8 | 4,52E-06 | Strong |
|  | 90 | 18417,7 | 2,14E-12 | Strong |
|  | 106 | 274 | 9,96E-10 | Strong |
|  | 114 | 1,1 | 7,23E-05 | Weak |
|  | 166 | 5,5 | 4,17E-09 | Moderate |
